# Bilateral globus pallidus interna deep brain stimulation in Parkinson’s disease: Therapeutic effects and motor outcomes prediction in a short-term follow up

**DOI:** 10.3389/fnhum.2022.1023917

**Published:** 2023-01-09

**Authors:** Dingding Shen, Linghao Cao, Yun Ling, Dianyou Li, Kang Ren, Weikun Shi, Zhonglue Chen, Haiyan Zhou, Jun Liu

**Affiliations:** ^1^Department of Neurology, Ruijin Hospital, Affiliated to Shanghai Jiao Tong University School of Medicine, Shanghai, China; ^2^Gyenno Science Co., Ltd., Shenzhen, China; ^3^Department of Neurosurgery, Ruijin Hospital, Affiliated to Shanghai Jiao Tong University School of Medicine, Shanghai, China; ^4^Co-innovation Center of Neuroregeneration, Nantong University, Nantong, China; ^5^HUST-GYENNO CNS Intelligent Digital Medicine Technology Center, Wuhan, China

**Keywords:** Parkinson’s disease, deep brain stimulation, globus pallidus interna, predictive factor, motor phenotype

## Abstract

**Objective:**

We aimed to compare the motor effect of bilateral globus pallidus interna (GPi) deep brain stimulation (DBS) on motor subtypes of Parkinson’s disease (PD) patients and identify preoperative predictive factors of short-term motor outcome.

**Methods:**

We retrospectively investigated bilateral GPi DBS clinical outcomes in 55 PD patients in 1 year follow up. Motor outcome was measured by the Movement Disorder Society Unified Parkinson’s Disease Rating Scale (MDS-UPDRS) part III before and 1 year after surgery. Clinical outcomes were compared among different motor subtypes. Preoperative predictors of motor outcome were assessed by performing univariate and multivariate linear regression and logistic regression analyses.

**Results:**

At 1 year following implantation, GPi DBS significantly improved the off-medication MDS-UPDRS III scores in all motor subtype cohorts, with prominent improvement in tremor. No significant difference of postoperative motor symptoms changes was found except greater tremor improvement achieved in both the tremor-dominant (TD) and indeterminate (IND) patients compared to the postural instability and gait difficulty (PIGD) patients. High percentage of PIGD patients were weak responders to DBS. Better levodopa responsiveness and more severe tremor predicted greater overall improvement of motor function in the entire cohort. Similarly, both levodopa responsiveness and tremor improvement were confirmed as predictors for motor improvement in PIGD patients.

**Conclusion:**

Bilateral GPi DBS could effectively improve motor outcomes in PD patients regardless of motor subtypes. Both TD and IND patients obtained larger tremor improvement. The intensity of levodopa responsiveness and the severity of tremor could serve as predictors of motor improvement 1 year after GPi DBS.

## Introduction

Deep brain stimulation (DBS) has evolved as an effective treatment option for management of medication-refractory motor fluctuations of Parkinson’s disease (PD) (Armstrong and Okun, 2020). Currently, the subthalamic nucleus (STN) and the globus pallidus interna (GPi) are two main targets for DBS. Increasing evidence across randomized clinical trials reported comparable motoric benefits between two targets (Anderson et al., 2005; Follett et al., 2010; Weaver et al., 2012; Odekerken et al., 2013), but slight target differences occur. Treatment with STN DBS usually yields a greater reduction in dopaminergic medications, while GPi DBS might offer greater benefits in dyskinesia, gait, and cognitive outcomes, as well as greater flexibility for medication adjustments (Ramirez-Zamora and Ostrem, 2018; Hartmann et al., 2019).

Based on motor symptoms, PD can be classified into tremor-dominant (TD), postural instability and gait difficulty (PIGD), and indeterminate (IND) subtypes (Stebbins et al., 2013; Thenganatt and Jankovic, 2014). Different PD motor subtypes have different neuroalterations (Boonstra et al., 2021) as well as different clinical courses. Compared to TD patients, PIGD patients typically demonstrate a worse disease course (Rajput et al., 2009; Arie et al., 2017). Besides, recent studies found significant difference in the responsiveness of motor subtypes to both GPi and STN DBS, with the TD motor subtype obtaining greater benefits compared to the PIGD patients, primarily due to greater tremor improvement (Katz et al., 2015; Tsuboi et al., 2020b). However, comparative motor outcomes among different PD motor phenotypes after GPi DBS remain insufficiently evaluated.

Accurate patient selection is a crucial determinant of favorable outcomes following DBS surgery (Pollak, 2013). Factors related to DBS outcomes are important for clinicians to predict the therapeutic effects in PD patients. Being considered as the best short-term postoperative motor response predictor, preoperative levodopa responsiveness has been routinely used to determine DBS candidacy (Lin et al., 2022). Other predictive factors of short-term motor response include TD phenotype, high off-medication UPDRS motor scores, young age at surgery, disease duration and baseline frontal lobe score. Since all these factors have been mostly studied in patients undergoing STN DBS, their predictive value concerning GPi DBS outcomes has remained unclear. To the best of our knowledge, only one recent study from our Functional Neurosurgery Center revealed the positive correlation between preoperative levodopa responsiveness and GPi DBS benefits, but in a small population (Lin et al., 2021).

In the present study, we sought to explore motor outcomes at 1 year following GPi DBS implantation in a large cohort of 55 PD patients. Changes in MDS-UPDRS III total and subscores were compared among different motor subtypes. Furthermore, we tried to identify preoperative predictors for the short-term motor outcome of GPi DBS in this cohort, especially in PIGD patients.

## Materials and methods

### Patients

We retrospectively studied 60 PD patients who recommended by neurologists and underwent bilateral GPi DBS at the Center for Functional Neurosurgery, Ruijin Hospital (Shanghai, China) from November 2016 to July 2019. All patients enrolled in the study have written informed consent, and the hospital ethics committee approved the study.

The inclusion criteria for surgery were as follows: (1) diagnosis of idiopathic PD based on the 2015 MDS-PD Criteria (Postuma et al., 2015); (2) response to levodopa following a preoperative levodopa challenge test; (3) disabling motor fluctuations or dyskinesia despite all drug strategies; (4) informed consent for the surgery; and (5) good general health and accommodation of regular postoperative programming and follow-ups. The exclusion criteria were as follows: (1) contraindication for neurosurgery or high-field magnetic resonance imaging (MRI); (2) severe dementia or neuropsychiatric disorders, and (3) organic cerebral abnormalities.

### Surgical procedure and programming

When selecting GPi or STN as the target, each patient’s profile was carefully evaluated including motor symptoms, non-motor symptoms and medications, especially severity of dyskinesias, dystonia, axial symptoms, cognition and mood problems (Ramirez-Zamora and Ostrem, 2018). All patients underwent 3.0 Tesla MRI before surgery. We applied the Leksell stereotactic frame to the patient’s head followed by a head CT scan. The specific target coordinates and trajectory were defined using the SurgiPlan system after the coregistration of MRI-CT images, targeting the posterior GPi. All patients were implanted bilaterally with DBS leads simultaneously (model 3,387; Medtronic, Minneapolis, MN, USA) under general anesthesia. The implantable pulse generators (Activa RC, Medtronic, Minneapolis, MN, USA) were placed subclavicularly and connected with electrodes *via* subcutaneous wires at the same day. Postoperative head CT scan was performed and the image study was fuse to the preoperative targeting MRI to confirm satisfactory electrode placement of DBS leads and absence of complications. Each patient was carefully programmed monthly within the first half year after surgery to achieve optimal stimulation settings, and was followed by regular visits every 3 months or as clinically indicated.

### Outcome measures

All patients underwent standardized evaluation preoperatively and about 1 year postoperatively. To reduce evaluation bias, all the motor assessments were videotaped by one evaluator and were independently scored by one experienced movement disorder specialist in a blinded condition excepted for the rigidity subscore was directly scored by the evaluator.

Preoperatively, a 12 h overnight withdrawal of dopaminergic medications was required to perform off-medication motor assessments based on the MDS-UPDRS III (Goetz et al., 2008). After taking medication for about 60 min, the patients underwent the on-medication motor assessments. Postoperatively, all patients were assessed under two conditions: off-medication on-stimulation (MedOff/StimOn) evaluations were performed following a 12 h overnight dopaminergic medication cessation and kept stimulation on. Off-medication off-stimulation (MedOff/StimOff) was performed subsequently after turning stimulation off for 1 h.

We extracted tremor total (items 3.15–3.18), rigidity total (item 3.3), bradykinesia total (items 3.2, 3.4–3.8, and 3.14), axial total (items 3.1, and 3.9–3.13), speech subscore (item 3.1), arising from chair subscore (item 3.9), gait subscore (item 3.10), freezing of gait (item 3.11) and postural stability subscore (item 3.12) from MDS-UPDRS III for further analyses. We also evaluated Hoehn–Yahr stage (H-Y) and MDS-UPDRS II and calculated levodopa equivalent daily dose (LEDD) (Tomlinson et al., 2010).

### Definition of motor subtypes

We calculated the ratio of the mean tremor score from MDS-UPDRS II and III (items 2.10, 3.15–3.18) to the mean PIGD score (items 2.12, 2.13, 3.10–3.12) to delineate TD patients (ratio ≥1.15), PIGD patients (ratio ≤0.90), and IND patients (ratios >0.9 and <1.15) (Stebbins et al., 2013).

### Statistical analysis

Demographical variables, motor variables at baseline and 1 year follow-up were described. Continuous variables were presented as mean ± SD, and categorical variables were presented as percentages and numbers. Chi-square test was used to test the difference of classifying variables between groups. Shapiro-Wilk test was used to test the normality of the distribution. For normally distributed data, independent *t*-test and Paired *t*-test were performed. For non-normally distributed data, Wilcoxon rank-sum test and Wilcoxon signed-rank test were performed. Independent *t*-test and Wilcoxon rank-sum test were used to test the difference of motor symptoms between groups. Paired *t*-test and Wilcoxon signed-rank test were used to test the improvement of motor symptoms. During the multiple testing, *p*-values were adjusted by Bonferroni correction.

The percentage change of total MDS-UPDRS part III score between baseline MedOff and MedOff/StimOn states at 1 year after GPi-DBS was considered as the postoperative independent variable for analyzing the effect of surgery. For discriminating the surgery effect, response type was defined. The percentage change of postoperative total MDS-UPDRS part III score between baseline MedOff and MedOff/StimOn states ≥24% was considered as good response, or otherwise considered as weak response (Merello et al., 2011). A variety of clinical factors collected before the surgery were selected as preoperative independent variables including sex, age at surgery, disease duration, age onset, EOPD (age onset <40 years), MMSE score, HAMD-17 score, H-Y stage in both of the MedOff and MedOn conditions, absolute changes and percentage changes of motor symptoms between the MedOff and MedOn states, L-dopa responsiveness, PD motor phenotype, MDS-UPDRS TD/PIGD score and LEDD.

Linear regression and logistic regression were performed based on the dependent variable of percentage change of postoperative total MDS-UPDRS part III score and response type, respectively. Univariate regression and multivariate regression were performed for analyzing the relationship between dependent variables and preoperative independent variables. In univariate and multivariate study, β from regression was used to interpret the relationship between surgical effect and preoperative variables. All potential variables were performed by univariate regression firstly, and the variables with significant β were selected for multivariate regression. During multivariate regression, Variance Inflation Factor (VIF) was used to detect the collinearity between variables. In order to avoid the collinearity problem, the variable with VIF greater than 10 was removed, and then rest of variables were performed by multivariate regression until VIFs of all variables were smaller than 10. Statistical significance was tested at two-sided with *p*-value < 0.05. All statistical analysis were performed using R.4.0.2 (R Core Team, Vienna, Austria).

## Results

### DBS outcomes of the entire cohort

We collected a total of 60 patients who underwent bilateral GPi DBS surgeries at our center. Five patients were excluded because of incomplete data or unsatisfactory electrodes placement. Consequently, the cohort comprised 55 patients who fulfilled the inclusion criteria. Their demographic information and baseline characteristics were shown in Table 1.

**TABLE 1:** Baseline characteristics of the whole cohort and different motor subtypes.

Characteristics	The whole cohort (*N* = 55)	PIGD (*N* = 38)	IND (*N* = 6)	TD (*N* = 11)
Sex (male, %)	32 (58.2%)	21 (55.3%)	3 (50%)	8 (72.7%)
Age at DBS (y)	64.6 ± 8.0	64.75 ± 8.17	68.9 ± 6.06	61.59 ± 7.85
Age at disease onset (y)	51.4 ± 7.7	51.32 ± 7.73	57.17 ± 4.92^a^*	48.73 ± 7.67
Disease duration (m)	159.8 ± 57.7	163.13 ± 59.06	144.17 ± 44.9	156.64 ± 61.91
Last follow-up (m)	12.1 ± 6.2	11.6 ± 5.69	13.47 ± 6.19	12.86 ± 8.05
L-dopa responsiveness, % of improvement	52 ± 17%	52 ± 12%	56 ± 19%	60 ± 17%
MMSE	27.6 ± 1.9	27.61 ± 1.94	27 ± 3.03	27.73 ± 1.35
LEDD	858.5 ± 283.5	855.45 ± 277.98	914 ± 301.51	838.59 ± 316.61
HAMD-17	9.1 ± 6.2	8.4 ± 6.6	10.7 ± 6.8	10.6 ± 4.4

^a^Indicates the statistical difference comparing to PIGD subtype.

*P-value < 0.05.

PIGD, postural instability and gait difficulty; TD, tremor dominant; IND, intermediate; MMSE, mini-mental state exam; LEDD, levodopa equivalent daily dose. HAMD, hamilton depression scale.

**FIGURE 1:**
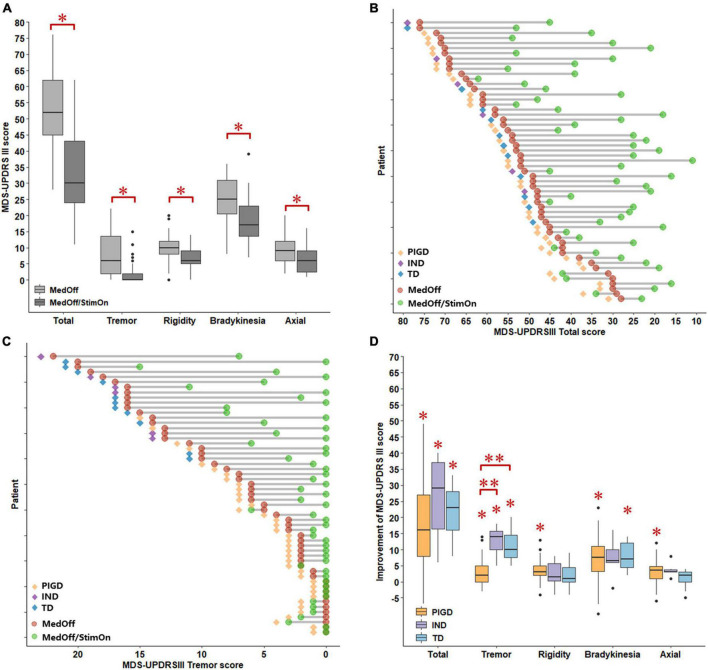
Comparisons of MDS-UPDRS III scores between baseline MedOff and MedOff/StimOn states in 1 year follow up **(A)**. Individual changes of MDS-UPDRS III scores between baseline MedOff (red point) and MedOff/StimOn (green point) states with PIGD (orange rhombus), IND (purple rhombus) and TD subtypes (blue rhombus) **(B)**. Individual changes of MDS-UPDRSIII tremor scores between baseline MedOff (red point) and MedOff/StimOn (green point) states with PIGD (orange rhombus), IND (purple rhombus) and TD subtypes (blue rhombus) **(C)**. Postoperative improvement of the MDS-UPDRS III between baseline MedOff and MedOff/StimOn for the patients with PIGD, IND, and TD subtypes **(D)**. For the boxplots of **(A,D)**, the central line depicted the median, the top and the bottom of the box indicated the 75th quantile (Q3) and 25th quantile (Q1), the top and the bottom of the error bar stated the “maximum” (Q3 + 1.5 × interquartile range) and the “minimum” (Q1–1.5 × interquartile range), dots represented the outliers. *Indicate significant differences between baseline MedOff and MedOff/StimOn states. ^**^Indicate significant differences between the two groups. MDS UPDRS-III, movement disorder society unified Parkinson’s disease rating scale-motor part; MedOff, preoperative off-medication state; MedOff/StimOn, postoperative off-medication/on-stimulation state; PIGD, postural instability and gait difficulty; IND, intermediate; TD, tremor dominant.

Pre- and postoperative motor outcomes were presented in Table 2. At 1 year after DBS implantation, MDS-UPDRS III total, tremor total, rigidity total, bradykinesia total, axial total, speech, gait, and freezing of gait and postural stability scores in the MedOff/StimOn state were all significantly improved compared with baseline MedOff state (Figure 1A). There was no significant change in arising from chair score and H-Y stage. Notably, improvement of tremor was the most prominent among the components of MDS-UPDRS III. Individual MDS-UPDRS III total scores were all improved after surgery except four PIGD patients (Figure 1B). Figure 1C demonstrated individual changes in tremor scores. All patients had some improvement of tremor except that six PIGD patients got worse tremor. There was no significant difference in MDS-UPDRS III total scores and subscores between postoperative MedOff/StimOff state and prepoperative MedOff state, except reduced tremor scores.

**TABLE 2:** Deep brain stimulation (DBS) outcomes of the whole cohort.

	Preoperation			Postoperation		
Motor symptom	MedOff	MedOn	Improvement (%)	MedOff	MedOff/StimOn	Improvement (%)
H-Y	2.6 ± 0.5	2.3 ± 0.4^a^*	0.3 ± 0.5 (8 ± 18%)	2.6 ± 0.5	2.5 ± 0.5	0.1 ± 0.5 (3 ± 20%)
MDS-UPDRSIII	–	–	–	–	–	–
Total score	52.4 ± 13.1	24.4 ± 8.5^a^**	28.0 ± 11.6 (52 ± 17%)	50.0 ± 13.2	33.5 ± 12.3^c^**^d^**	18.9 ± 13.4 (34 ± 25%)
Tremor total	7.5 ± 6.8	1.2 ± 2.2^a^**	6.3 ± 6.4 (75 ± 35%)	5.7 ± 7.1^b^*	1.9 ± 3.1^c^**^d^**	5.6 ± 5.8 (79 ± 29%)
Rigidity total	9.8 ± 4.0	5.0 ± 3.6^a^**	4.8 ± 3.0 (50 ± 29%)	9.6 ± 3.7	6.7 ± 3.6^c^**^d^**	3.1 ± 3.7 (26 ± 49%)
Bradykinesia total	25.6 ± 6.3	14.0 ± 4.6^a^**	11.6 ± 5.8 (43 ± 20%)	25.8 ± 6.1	18.3 ± 6.7^c^**^d^**	7.3 ± 7 (25 ± 32%)
Axial total	9.4 ± 4.2	4.2 ± 3.0^a^**	5.3 ± 3.2 (55 ± 25%)	8.9 ± 4.1	6.6 ± 4.0^c^**^d^**	2.8 ± 3.9 (26 ± 46%)
Speech	1.4 ± 1.0	0.9 ± 0.7^a^**	0.5 ± 0.6 (33 ± 36%)	1.5 ± 0.9	1.1 ± 0.8^c^**^d^*	0.4 ± 0.9 (26 ± 52%)
Arising from chair	0.95 ± 1.1	0.07 ± 0.26^a^**	0.9 ± 1.02 (94 ± 20%)	1.7 ± 0.7	1.2 ± 0.8^c^*	0.3 ± 1 (50 ± 65%)
Gait	1.8 ± 0.8	0.7 ± 0.6^a^**	1.2 ± 0.8 (62 ± 34%)	1.7 ± 0.7	1.2 ± 0.8^c^**^d^**	0.6 ± 0.9 (32 ± 51%)
Freezing of gait	1.2 ± 1.3	0.3 ± 0.6^a^**	0.9 ± 1.2 (76 ± 36%)	1.2 ± 1.2	0.8 ± 1.0^c^**^d^*	0.4 ± 1.2 (45 ± 56%)
Postural stability	2.3 ± 1.4	1.4 ± 1.4^a^**	0.9 ± 1.3 (42 ± 64%)	2.3 ± 1.5	1.9 ± 1.5^c^*^d^*	0.4 ± 1.5 (9 ± 82%)

Values are presented as means ± SDs.

^a^Indicates the statistical difference of the MDS UPDRS-III score and subscores between the MedOff and MedOn states.

^b^Indicates the statistical difference of the MDS UPDRS-III score and subscores between the MedOff and MedOff/StimOff states.

^c^Indicates the statistical difference of the MDS UPDRS-III score and subscores between the MedOff/StimOff and MedOff/StimOn states.

^d^Indicates the statistical difference of the MDS UPDRS-III score and subscores between the MedOff and MedOff/StimOn states.

*P-value < 0.05. **p-value < 0.01.

MDS UPDRS-III, movement disorder society unified Parkinson’s disease rating scale-motor part; MedOff, preoperative off-medication state; MedOn, preoperative on-medication state; MedOff/StimOff, postoperative off-medication/off-stimulation state; MedOff/StimOn, postoperative off-medication/on-stimulation state.

### PIGD versus non-PIGD subtypes

As illustrated in Table 1, 38, 6 and 11 patients were classified as having the PIGD, IND, and TD subtypes, respectively. Compared with PIGD patients, IND patients had older age at disease onset. There was no difference in demographics found by the PIGD and TD subtypes. At baseline as shown in Table 3, both TD and IND patients had worse tremor scores than PIGD patients in the off-medication state. In addition, PIGD patients had worse axial symptoms such as worse H-Y stage (2.7 ± 0.5 vs. 2.0 ± 0.2, *p* < 0.001), axial total score (10.2 ± 4.4 vs. 6.2 ± 3.1, *p* < 0.001) and postural stability (2.6 ± 1.4 vs. 0.9 ± 0.5, *p* < 0.001) compared to the TD group. In the on-medication state, no significant difference was found among three groups.

**TABLE 3:** Baseline characteristics of the patients with different motor subtypes.

Motor symptom	PIGD (*N* = 38)			IND (*N* = 6)			TD (*N* = 11)		
	MedOff	MedOn	Improvement (%)	MedOff	MedOn	Improvement (%)	MedOff	MedOn	Improvement (%)
HY	2.7 ± 0.5	2.4 ± 0.5^a^*	0.3 ± 0.6 (11 ± 19%)	2.7 ± 0.4	2.3 ± 0.4	0.3 ± 0.3 (12.2 ± 9.6%)	2 ± 0.2^b^**	2.1 ± 0.3	0 ± 0.4 (−2.7 ± 16.8%)
MDS-UPDRSIII	–	–	–	–	–	–	–	–	–
Total score	50.3 ± 14.0	24.6 ± 7.1^a^**	25.7 ± 11.8 (49 ± 17%)	61 ± 10.7	27 ± 12.4^a^**	34 ± 11.2 (56.2 ± 18.7%)	54.7 ± 8.7	22.1 ± 10.9^a^**	32.6 ± 9.1 (60.4 ± 17%)
Tremor total	3.7 ± 3.9	0.7 ± 1.4^a^**	3.0 ± 4.0 (69 ± 42%)	16.3 ± 3.4^b^**	2.8 ± 3.3	13.5 ± 3.9^c^** (83.4 ± 19.6%)	15.7 ± 3.4^b^**	2 ± 3^a^*	13.7 ± 4.4^c^** (87.5 ± 18.1%)
Rigidity total	10.4 ± 4.3	5.5 ± 3.6^a^**	4.9 ± 3.0 (48 ± 26%)	9.3 ± 3.4	5.2 ± 3.7	4.2 ± 2.5 (47.5 ± 31.2%)	8.3 ± 2.8	3.4 ± 3.4^a^**	4.9 ± 3.1 (62.9 ± 37.7%)
Bradykinesia total	26.0 ± 6.9	13.8 ± 4.0^a^**	12.1 ± 6.0 (44 ± 21%)	25.2 ± 5.2	14.7 ± 5.9^a^*	10.5 ± 6.5 (40.7 ± 22.3%)	24.5 ± 4.8	14 ± 6.2^a^**	10.5 ± 4.8 (43.8 ± 22.1%)
Axial total	10.2 ± 4.4	4.6 ± 3.1^a^**	5.7 ± 3.5 (55 ± 26%)	10.2 ± 1.8	4.3 ± 2.6^a^**	5.8 ± 2.1 (58.5 ± 22.5%)	6.2 ± 3.1^b^**	2.7 ± 2.6^a^*	3.5 ± 2.1 (58.1 ± 28.3%)
Speech	1.4 ± 1.0	0.9 ± 0.7^a^**	0.6 ± 0.7 (38 ± 36%)	1.2 ± 1.2	0.8 ± 1	0.3 ± 0.5 (33.3 ± 47.1%)	1.5 ± 0.7	1.2 ± 0.6	0.4 ± 0.5 (21.2 ± 33.4%)
Arising from chair	1.11 ± 1.25	0.11 ± 0.31^a^**	1 ± 1.16 (91 ± 24%)	1 ± 0	0 ± 0^a^**	1 ± 0 (100 ± 0%)	0.4 ± 0.5	0 ± 0^a^**	0.4 ± 0.5 (100 ± 0%)
Gait	1.9 ± 0.9	0.7 ± 0.6^a^**	1.2 ± 0.8 (61 ± 33%)	2 ± 0	0.7 ± 0.5	1.3 ± 0.5 (66.7 ± 25.8%)	1.5 ± 0.5	0.5 ± 0.7^a^*	0.9 ± 0.7 (63.6 ± 45.2%)
Freezing of gait	1.4 ± 1.4	0.3 ± 0.6^a^**	1.0 ± 1.3 (70 ± 39%)	0.8 ± 1	0 ± 0	0.8 ± 1 (100 ± 0%)	0.6 ± 0.9	0.1 ± 0.3	0.5 ± 0.8 (87.5 ± 25%)
Postural stability	2.6 ± 1.4	1.6 ± 1.6^a^**	1.0 ± 1.4 (42 ± 60%)	2.7 ± 1.2	1.7 ± 1.4	1 ± 0.6 (47.2 ± 32.3%)	0.9 ± 0.5^b^**	0.5 ± 0.9	0.4 ± 0.9 (38.9 ± 99.3%)

Values are presented as means ± SDs.

^a^Indicates the statistical difference of the MDS UPDRS-III score and subscores between the MedOff and MedOn states.

^b^Indicates the statistical difference of the MDS UPDRS-III score and subscores in MedOff or MedOn states comparing to PIGD subtype.

^c^Indicates the statistical difference of the improvement or improvement percentage of MDS UPDRS-III score and subscores comparing to PIGD subtype.

*P-value < 0.05. **p-value < 0.01.

MDS UPDRS-III, movement disorder society unified Parkinson’s disease rating scale-motor part; PIGD, postural instability and gait difficulty; TD, tremor dominant; IND, intermediate; MedOff, preoperative off-medication state; MedOn, preoperative on-medication state.

After surgery, as shown in Table 4 and Figure 1D, The PIGD patients exhibited substantial improvement in the MDS-UPDRS III total and all subscores except H-Y stage and arising from chair compared to baseline MedOff states. The TD patients experienced significant improvement in the MDS-UPDRS III total, tremor total and bradykinesia total scores. The IND patients also had significant improvement in the MDS-UPDRS III total and tremor total scores. When compared with PIGD patients, no clear differences in postoperative changes were identified in both TD and IND patients except larger response of tremor. Mean tremor total improvement scores were greater in the TD and IND patients (11.2 ± 4.6 vs. 2.9 ± 4.0, *p* < 0.001; 12.7 ± 4.8 vs. 2.9 ± 4.0, *p* < 0.001, respectively).

**TABLE 4:** Deep brain stimulation (DBS) outcomes of different motor subtypes.

	PIGD (*N* = 38)			IND (*N* = 6)			TD (*N* = 11)		
Motor symptom	MedOff	MedOff/StimOn	Improvement (%)	MedOff	MedOff/StimOn	Improvement (%)	MedOff	MedOff/StimOn	Improvement (%)
H-Y	2.7 ± 0.5	2.5 ± 0.5	0.2 ± 0.5 (7 ± 18%)	2.7 ± 0.4	2.4 ± 0.5	0.2 ± 0.3 (9 ± 10%)	2 ± 0.2^b^**	2.3 ± 0.5	−0.3 ± 0.5 (−14 ± 25%)
MDS-UPDRSIII	–	–	–	–	–	–	–	–	–
Total score	50.3 ± 14.0	33.5 ± 12.6^a^**	16.8 ± 14.2 (31 ± 27%)	61 ± 10.7	35 ± 13.9 ^a^*	26 ± 13.9 (42 ± 22%)	54.7 ± 8.7	32.6 ± 11.3^a^**	22.1 ± 7.9 (41 ± 16%)
Tremor total	3.7 ± 3.9	0.8 ± 1.5^a^**	2.9 ± 4.0 (82 ± 32%)	16.3 ± 3.4^b^**	3.7 ± 4.6^a^*	12.7 ± 4.8^c^** (78 ± 28%)	15.7 ± 3.4^b^**	4.5 ± 4.5^a^**^b^*	11.2 ± 4.6^c^** (72 ± 25%)
Rigidity total	10.4 ± 4.3	6.8 ± 3.8^a^**	3.6 ± 3.6 (32 ± 37%)	9.3 ± 3.4	7 ± 4.8	2.3 ± 4.5 (23 ± 49%)	8.3 ± 2.8	6.2 ± 2.5	2.1 ± 3.6 (7 ± 75%)
Bradykinesia total	26.0 ± 6.9	18.8 ± 7.1^a^**	7.2 ± 7.9 (23 ± 36%)	25.2 ± 5.2	17.8 ± 5.6	7.3 ± 6 (28 ± 23%)	24.5 ± 4.8	16.8 ± 6^a^*	7.7 ± 4.4 (32 ± 18%)
Axial total	10.2 ± 4.4	7.1 ± 4.4^a^**	3.2 ± 4.2 (27 ± 45%)	10.2 ± 1.8	6.5 ± 2.1	3.7 ± 2.3 (36 ± 18%)	6.2 ± 3.1^b^*	5.1 ± 3.3	1.1 ± 2.9 (15 ± 61%)
Speech	1.4 ± 1.0	1.0 ± 0.8^a^**	0.4 ± 0.9 (31 ± 51%)	1.2 ± 1.2	1 ± 1.1	0.2 ± 1 (4 ± 48%)	1.5 ± 0.7	1.2 ± 0.8	0.4 ± 0.8 (17 ± 55%)
Arising from chair	1.11 ± 1.25	0.76 ± 1	0.3 ± 1.1 (54 ± 63%)	1 ± 0	0.3 ± 0.5	0.7 ± 0.5 (67 ± 52%)	0.4 ± 0.5	0.5 ± 0.7	−0.1 ± 0.5 (0 ± 82%)
Gait	1.9 ± 0.9	1.3 ± 0.8^a^**	0.6 ± 0.9 (30 ± 50%)	2 ± 0	1.2 ± 0.4	0.8 ± 0.4 (42 ± 20%)	1.5 ± 0.5	0.9 ± 0.9	0.5 ± 0.9 (36 ± 67%)
Freezing of gait	1.4 ± 1.4	0.9 ± 1.1^a^*	0.5 ± 1.4 (55 ± 49%)	0.8 ± 1	0.7 ± 1	0.2 ± 1 (0 ± 100%)	0.6 ± 0.9	0.5 ± 0.8	0.1 ± 0.7 (25 ± 50%)
Postural stability	2.6 ± 1.4	2.0 ± 1.5^a^*	0.6 ± 1.6 (23 ± 50%)	2.7 ± 1.2	2 ± 1.7	0.7 ± 0.5 (39 ± 38%)	0.9 ± 0.5^b^**	1.4 ± 1.4	−0.5 ± 1.4 (−67 ± 141%)

Values are presented as means ± SDs.

^a^Indicates the statistical difference of the MDS UPDRS-III score and subscores between the MedOff and MedOff/StimOn states.

^b^Indicates the statistical difference of the MDS UPDRS-III score and subscores in MedOff or MedOff/StimOn states comparing to PIGD subtype.

^c^Indicates the statistical difference of the improvement or improvement percentage of MDS UPDRS-III score and subscores comparing to PIGD subtype.

*P-value < 0.05. **p-value < 0.01.

MDS UPDRS-III, movement disorder society unified Parkinson’s disease rating scale-motor part; PIGD, postural instability and gait difficulty; TD, tremor dominant; IND, intermediate; MedOff, preoperative off-medication state; MedOff/StimOn, postoperative off-medication/on-stimulation state.

### Preoperative predictors of GPi DBS outcome in the entire cohort

As illustrated in Table 5, univariate linear regression analyses identified the following preoperative clinical factors predictive of short-term motor response: L-dopa responsiveness, off-medication tremor score, gait improvement, arising from chair improvement, off-medication gait score, off-medication axial score, bradykinesia improvement percentage, tremor improvement percentage. Multivariate linear regression model confirmed that levodopa responsiveness and off-medication tremor score were independent preoperative predictors of greater short-term motor improvement after surgery. The Pearson correlation coefficient of this model was 0.6632, and the adjusted R-Squared was 0.3699.

**TABLE 5:** Linear regression analysis identified factors influencing short-term motor outcome in the entire cohort.

	Univariate analysis			Multivariate analysis		
Baseline variable	β	95% CI	*P*	β	95% CI	*P*
L-dopa responsiveness, % of improvement	0.499	0.260–0.738	<0.001	3.871	0.34–7.4	0.032
MedOff tremor	0.407	0.155–0.659	0.002	4.280	1.13–7.43	0.009
Gait improvement	0.347	0.088–0.605	0.009	−0.255	−5.15 to 4.64	0.917
Arising from chair improvement	0.414	0.164–0.665	0.002	3.124	−1.49 to 7.74	0.180
MedOff gait	0.382	0.127–0.637	0.004	3.265	−2.27 to 8.8	0.241
MedOff axial	0.318	0.057–0.579	0.018	−0.767	−5.83 to 4.3	0.762
Bradykinesia improvement percentage	0.429	0.181–0.678	0.001	Removed by collinearity checking		
Tremor improvement percentage	0.530	0.260–0.800	<0.001	Removed by collinearity checking		

MDS UPDRS-III, movement disorder society unified Parkinson’s disease rating scale-motor part; MedOff, preoperative off-medication state.

When we created two cohorts with favorable (good responder) and unfavorable (weak responder) outcomes, it was noted that 14 out of 17 patients with unfavorable outcomes were PIGD patients and only one was a TD subtype. Among 14 PIGD weak responders, motor function of four patients was even worse than pre-operation (Figure 1B). There was no significant difference in MDS-UPDRS III total and subscores between the two subgroups in both off-medication and on-medication state, except that the good responders had longer disease duration (169.9 ± 58.2 vs. 137.2 ± 50.9 months, *p* = 0.043) and greater tremor improvement (7.8 ± 6.5 vs. 3.1 ± 4.7, *p* = 0.0424) (Supplementary Tables 1, 2).

As illustrated in Table 6, preoperative predictors of favorable outcomes of logistic regression analysis included L-dopa responsiveness, off-medication H-Y stage, axial improvement, MDS-UPDRSIII total improvement, tremor improvement percentage, tremor improvement and off-medication tremor score. Levodopa responsiveness was confirmed as the only independent preoperative predictor of favorable response in multivariate linear regression model.

**TABLE 6:** Logistic regression analysis identified factors influencing short-term Motor outcome in the entire cohort.

	Univariate analysis			Multivariate analysis		
Baseline variable	β	95% CI	*P*	β	95% CI	*P*
L-dopa responsiveness, % of improvement	0.178	0.062–0.294	0.004	0.198	0.033–0.362	0.022
MedOff HY stage	−0.132	−0.252 to −0.011	0.037	−0.114	−0.256 to 0.028	0.122
Axial improvement	0.125	0.004–0.246	0.049	−0.080	−0.270 to 0.110	0.413
MDS-UPDRSIII total improvement	0.168	0.051–0.285	0.007	Removed by collinearity checking		
Tremor improvement percentage	0.170	0.048–0.292	0.009	Removed by collinearity checking		
Tremor improvement	0.160	0.042–0.278	0.010	Removed by collinearity checking		
MedOff Tremor	0.146	0.027–0.265	0.020	Removed by collinearity checking		

MDS UPDRS-III, movement disorder society unified Parkinson’s disease rating scale-motor part.

### Preoperative predictors of GPi DBS in PIGD subtypes

For motor response in PIGD group, univariate linear regression analyses identified the following preoperative clinical factors predictive of short-term motor response: tremor percentage improvement (beta = 0.63, *p*-value < 0.01), tremor improvement (beta = 0.56, *p*-value < 0.01), levodopa responsiveness (beta = 0.66, *p*-value < 0.01), and MDS-UPDRSIII total improvement (beta = 0.69, *p*-value < 0.01). Multivariate linear regression model confirmed that both levodopa responsiveness (beta = 4.4250, *p*-value = 0.0413) and tremor improvement (beta = 4.5921, *p*-value = 0.0487) were independent preoperative predictors. The Pearson correlation coefficient of this model was 0.7352, and the adjusted R-Squared was 0.4856.

When we discriminated PIGD patients with favorable and unfavorable outcomes, it was noted that 37% (14 out of 38) PIGD patients were weak responders. There was no significant difference in MDS-UPDRS III total and subscores between the two subgroups in both off-medication and on-medication state, except that the good responders had longer disease duration (179.4 ± 58 vs. 135.3 ± 51.5 months, *p* = 0.021) and greater levodopa response (54 ± 12 vs. 40 ± 20%, *p* = 0.023) (Supplementary Tables 3, 4).

Univariate logistic regression analysis identified several preoperative predictors of favorable outcomes: levodopa responsiveness (beta = 0.21, *p*-value < 0.01), axial improvement (beta = 0.18, *p*-value = 0.019), tremor percentage improvement (beta = 0.20, *p*-value = 0.023), disease duration (beta = 0.18, *p*-value = 0.024), axial percentage improvement (beta = 0.18, *p*-value = 0.025), MDS-UPDRSIII total improvement (beta = 0.17, *p*-value = 0.032), tremor improvement (beta = 0.17, *p*-value = 0.033), postural stability improvement (beta = 0.16, *p*-value = 0.040). However, multivariate logistic regression analysis only confirmed levodopa responsiveness (beta = 0.20, *p*-value = 0.049) as the independent predictor.

### DBS programming and medications

For the entire cohort, the mean ± SD of the stimulation settings at 1 year were as follows: voltage (left, 3.3 ± 0.7 V; right, 3.0 ± 0.8 V), pulse width (left, 83.4 ± 13.6 μs; right, 81.4 ± 13.6 μs), and frequency (147.8 ± 27.9 Hz) (Supplementary Table 5).

For the whole cohort, there was a significant decrease in LEDD after DBS compared to baseline (813.1 ± 280.8 vs. 658.9 ± 282.6 mg, *p* < 0.001). The TD patients had significantly larger change in LEDD compared with the PIGD patients (TD, 35.2 ± 13.6%; PIGD, 8.4 ± 33.1%, *p* = 0.002) (Supplementary Table 6).

## Discussion

The current study is the large retrospective study to evaluate short term effects of bilateral GPi DBS in Asian PD patients. First of all, we found that patients in both TD and IND groups experienced greater tremor improvement after DBS compared with patients in the PIGD group. Second, both preoperative levodopa responsiveness and the severity of tremor predicted the motor function after GPi DBS in general. Third, both preoperative levodopa responsiveness and tremor improvement served as the predictors of motor function improvement in PIGD subtypes.

At 1 year after GPi implantation, we observed improvement of MDS-UPDRS III total scores in the MedOff/StimOn state by 18.9 points compared to baseline MedOff state. This finding was congruent with previous trials reporting an overall improvement of 11.4–12.6 point (Follett et al., 2010; Odekerken et al., 2013; Tsuboi et al., 2020b). No significant difference was found between the MedOff/StimOff state and preoperative baseline in our cohort except the tremor score was reduced in the MedOff/StimOff state which was also observed in another cohort (Lin et al., 2021). Both enhanced stress-related tremors in preoperative MedOff state and the postoperative microlesions effect (Mann et al., 2009) could lead to the improvement of tremor score after DBS. Consistent with earlier findings (Anderson et al., 2005; Follett et al., 2010; Odekerken et al., 2013; Tsuboi et al., 2020b; Lin et al., 2021), GPi DBS significantly improved all cardinal symptoms such as tremor, rigidity, bradykinesia, and axial symptoms in our cohort. Individual changes in tremor scores in our cohort supported GPi DBS as an effective treatment for tremor (Tsuboi et al., 2020b).

To our knowledge, there are few studies evaluating the effects of GPi DBS on different PD motor subtypes. Our present study revealed that compared with the PIGD patients, the TD and IND patients experienced greater improvement in tremor. These results were comparable to previous findings reporting that TD patients had greater improvement in UPDRS III compared with the PIGD patients, where the biggest difference between the two subtypes was observed in tremor rather than axial symptoms (Katz et al., 2015; Tsuboi et al., 2020b). Indeed, in our patient cohort, different subtypes had similarly significant response to stimulation with respect to axial symptoms in a short term. In accordance with earlier studies (Katz et al., 2015; Lin et al., 2021), this finding indicates that GPi DBS may be efficacious and safe for PIGD patients as well as TD patients. Nevertheless, the classification of patients based on surgery effects revealed that a considerable part of PIGD patients were weak responders. DBS stimulation did not improve the motor function in four PIGD patients. This suggested that although PD patients had a favorable response to GPi DBS in general, individual difference did exist in PIGD cases. For clinicians, it is crucial to evaluate the variables that may influence the clinical outcomes of surgery in PIGD patients to identify the good candidate. PIGD patients with better levodopa response or greater tremor improvement may benefit more from GPi DBS as our study suggested.

Not surprisingly, preoperative levodopa responsiveness served as a predictor with short-term benefit after GPi DBS in our present study. The predictive effect of levodopa responsiveness in short-term good outcome after DBS has been confirmed by plenty of research (Su et al., 2017; Cavallieri et al., 2021; Lin et al., 2021). It is believed that motor symptoms that are not responsive to preoperative levodopa challenge are unlikely to improve with DBS. However, most previous studies have been focused on STN DBS. To the best of our knowledge, only one recent study confirmed the correlation between the preoperative levodopa responsiveness and GPi DBS responsiveness (Lin et al., 2021), but in a small population. Our study further provides strong support that this is the case in GPi DBS. Besides preoperative levodopa responsiveness, off-medication tremor was also identified as an independent predictor for overall PD patients, which was partly in line with previous studies enrolled STN DBS that TD subtype can predict short-term outcome (Cavallieri et al., 2021). Although more severe tremor is likely to occur in TD subtypes, only 20% TD subtypes in our cohort could significantly affect the statistical power. Similarly, in PIGD subtypes, both levodopa responsiveness and tremor improvement could predict better motor outcomes. Interestingly, it was found that good responders had longer disease duration in PIGD subtypes. Better levodopa responsiveness, greater tremor improvement and longer disease duration might imply more benign disease process in PIGD subtypes.

With respect to programming parameters, similar frequencies and pulse widths were utilized in our cohort as previous reports (Anderson et al., 2005; Follett et al., 2010; Odekerken et al., 2013; Tsuboi et al., 2020b). Traditionally, patients treated with STN DBS medication showed a significant reduction in LEDD postoperatively, while GPi DBS led to a more modest reduction. Notably, we observed significant LEDD reduction after GPi DBS, which was inconsistent with prior studies (Weaver et al., 2009; Tsuboi et al., 2020a,b). This discrepancy is possibly due to excellent levodopa responsiveness in our cohort, where 84% patients had levodopa responsiveness of above 40%, and 60% patients had levodopa responsiveness of above 50%. Tremor also contribute to significant LEDD reduction since TD patients in our cohort had much more LEDD reduction than PIGD subtypes. Although previous studies traditionally favored STN DBS compared to GPi target in respect to LEDD reduction, our study argued that GPi DBS could also lead to significant medication reduction. Considering its effective suppression of tremor and reduction of LEDD, GPi DBS may also be a good option for TD subtypes or those with full levodopa responsiveness who suffered medication-related adverse effects such as impulse control disorders, hallucination or excessive daytime sleepiness.

Several limitations should temper the strength of these results. The main drawback of our study was its retrospective character, although we have systemically evaluated preoperative features and postoperative changes in all subjects. Second, the small sample size of TD patients limited the statistical power. Third, the clinical preoperative factors incorporated in our prediction model are limited. In the future, the accuracy of the prediction model could be improved if other potential variables such as non-motor function including comprehensive neuropsychological assessment, brain MRI and even functional brain connectivity are included.

## Conclusion

Bilateral GPi DBS effectively improved overall motor outcomes in the entire PD cohort at 1 year follow-up, while TD and IND patients obtained greater tremor benefit from stimulation than PIGD patients. There was high percentage of weak responders in PIGD patients. For preoperative counseling in PIGD patients, evaluation of variables including age at disease onset, disease duration and progression rate was highly recommended. In general, preoperative levodopa responsiveness and the severity of tremor were predictable of motor outcome after GPi DBS in the short term. We expect that larger and prospective studies will explore motor and non-motor GPi DBS benefits across different motor subtypes in the long term, as well as predictors for the outcome of GPi DBS.

## Data availability statement

The original contributions presented in this study are included in the article/Supplementary material, further inquiries can be directed to the corresponding authors.

## Ethics statement

The studies involving human participants were reviewed and approved by the Ethics Committee of Ruijin hospital affiliated to Shanghai Jiao Tong University School of Medicine. The patients/participants provided their written informed consent to participate in this study.

## Author contributions

JL, HZ, and ZC: study conception and design, interpretation of data, and manuscript revision. DS, LC, and YL: study design, statistical analyses, interpretation of data, drafting, and manuscript revision. DL, KR, and WS: statistical analyses, interpretation of data, and review of manuscript. All authors read and approved the final version of the manuscript.
