# Supplementary material for: Bilateral globus pallidus interna deep brain stimulation in Parkinson’s disease: Therapeutic effects and motor outcomes prediction in a short-term follow up
Source: Front Hum Neurosci. 2023 Jan 9;16:1023917. doi: 10.3389/fnhum.2022.1023917 (PMC9868647; doi:10.3389/fnhum.2022.1023917)
Supplement: Supplementary file 1 [file Data_Sheet_1.docx]

**Supplemental Table 1. Demographic characteristics of the patients with diﬀerent DBS response.**

| **Characteristics** | **Good responders**  **(N=38)** | **Weak responders**  **(N=17)** | P_value | |
| --- | --- | --- | --- | --- |
| **Sex (Male, %)** | 21(55.3%) | 11(64.7%) | 0.719 |  |
| **Age at DBS (y)** | 64.6 ± 8.5 | 64.5 ± 7.2 | 0.946 |  |
| **Age at disease onset (y)** | 50.6 ± 7.8 | 53.2 ± 7.4 | 0.243 |  |
| **Disease duration (m)** | 169.9 ± 58.2 | 137.2 ± 50.9 | 0.043 |  |
| **Last follow-up (m)** | 12.4 ± 6.8 | 11.3 ± 4.4 | 0.486 |  |
| **MMSE** | 27.7 ± 1.9 | 27.2 ± 2.1 | 0.436 |  |
| **LEDD** | 838.5 ± 285.8 | 903 ± 281.4 | 0.441 |  |
| **HAMD-17** | 9.8 ± 6.7 | 7.4 ± 5.0 | 0.132 |  |

PIGD, Postural Instability and Gait Diﬃculty; MMSE, Mini-Mental State Exam; LEDD, Levodopa equivalent daily dose. HAMD, Hamilton Depression Scale.

**Supplemental Table 2. Baseline motor characteristics of the patients with diﬀerent DBS response.**

|  | **Good responders**  **(N=38)** | | | **Weak responders**  **(N=17)** | | |
| --- | --- | --- | --- | --- | --- | --- |
| **Motor symptom** | **MedOff** | **MedOn** | **Improvement**  **(%)** | **MedOff** | **MedOn** | **Improvement**  **(%)** |
| **HY** | 2.2 ± 0.4 | 2.6 ± 0.5 ^a*^ | 0.3 ± 0.6  (10 ± 19%) | 2.6 ± 0.5 | 2.5 ± 0.4 | 0.1 ± 0.4  (4 ± 17%) |
| **MDS-UPDRSIII** |  |  |  |  |  |  |
| Total score | 54.4 ± 12.6 | 23.6 ± 8.7 ^a**^ | 30.8 ± 9.6  (56 ± 13%) | 47.8 ± 13.4 | 25.9 ± 8.2 ^a**^ | 21.8 ± 13.4  (42 ± 22%) |
| Tremor total | 8.9 ± 6.9 | 1.2 ± 2.2 ^a**^ | 7.8 ± 6.5  (84 ± 28%) | 4.4 ± 5.4 | 1.3 ± 2 | 3.1 ± 4.7 ^b*^  (52 ± 46%) |
| Rigidity total | 9.4 ± 3.7 | 4.6 ± 3.5 ^a**^ | 4.8 ± 2.7  (55 ± 29%) | 10.9 ± 4.5 | 6.1 ± 3.6 ^a**^ | 4.8 ± 3.5  (42 ± 30%) |
| Bradykinesia total | 26.4 ± 5.5 | 13.9 ± 4.5 ^a**^ | 12.5 ± 4.6  (47 ± 15%) | 23.8 ± 7.8 | 14 ± 5 ^a**^ | 9.8 ± 7.6  (36 ± 29%) |
| Axial total | 9.7 ± 4.5 | 4 ± 3.2 ^a**^ | 5.7 ± 3.2  (60 ± 24%) | 8.8 ± 3.6 | 4.6 ± 2.6 ^a**^ | 4.2 ± 3  (45 ± 27%) |
| Speech | 1.5 ± 1 | 0.9 ± 0.7 ^a**^ | 0.6 ± 0.7  (37 ± 38%) | 1.3 ± 0.8 | 0.9 ± 0.7 | 0.4 ± 0.5  (24 ± 32%) |
| Arising from chair | 1.1 ± 1.2 | 0.1 ± 0.3 ^a**^ | 1 ± 1.1  (94 ± 21%) | 0.7 ± 0.9 | 0.1 ± 0.2 | 0.6 ± 0.9  (94 ± 18%) |
| Gait | 1.9 ± 0.8 | 0.7 ± 0.6 ^a**^ | 1.2 ± 0.9  (64 ± 36%) | 1.7 ± 0.7 | 0.7 ± 0.5 ^a**^ | 1 ± 0.6  (57 ± 31%) |
| Freezing of gait | 1.3 ± 1.3 | 0.3 ± 0.6 ^a**^ | 1 ± 1.2  (76 ± 36%) | 0.9 ± 1.4 | 0.2 ± 0.5 | 0.7 ± 1.2  (75 ± 42%) |
| Postural stability | 2.2 ± 1.4 | 1.2 ± 1.4 ^a**^ | 1 ± 1.3  (51 ± 60%) | 2.4 ± 1.4 | 1.8 ± 1.4 | 0.6 ± 1  (23 ± 73%) |

Values are presented as means±SDs.

***a*** indicates the statistical difference of the MDS UPDRS-III score and subscores between the MedOff and MedOn states.

***b*** indicates the statistical difference of the improvement or improvement percentage of MDS UPDRS-III score and subscores between the two groups.

**p* value < 0.05. ***p* value < 0.01.

MDS UPDRS-III, Movement Disorder Society Uniﬁed Parkinson’s Disease Rating Scale-Motor Part; PIGD, Postural Instability and Gait Diﬃculty; MedOff, preoperative off-medication state; MedOn, preoperative on-medication state.

**Supplemental Table 3. Demographic characteristics of the PIGD patients with diﬀerent DBS response.**

| **Characteristics** | **Good responders**  **(N=24)** | **Weak responders**  **(N=14)** | P_value | |
| --- | --- | --- | --- | --- |
| **Sex (Male, %)** | 12(50%) | 9(64.3%) | 0.606 |  |
| **Age at DBS (y)** | 65.2 ± 8.8 | 63.9 ± 7.3 | 0.618 |  |
| **Age at disease onset (y)** | 50.5 ± 7.6 | 52.8 ± 8 | 0.385 |  |
| **Disease duration (m)** | 179.4 ± 58 | 135.3 ± 51.5 | 0.021 |  |
| **Last follow-up (m)** | 11.6 ± 6.3 | 11.7 ± 4.6 | 0.954 |  |
| **MMSE** | 27.6 ± 2.2 | 27.6 ± 1.5 | 0.929 |  |
| **LEDD** | 855.7 ± 278.5 | 855.1 ± 287.7 | 0.995 |  |
| **HAMD-17** | 9.3 ± 7.5 | 6.9 ± 4.9 | 0.538 |  |

PIGD, Postural Instability and Gait Diﬃculty; MMSE, Mini-Mental State Exam; LEDD, Levodopa equivalent daily dose. HAMD, Hamilton Depression Scale.

**Supplemental Table 4. Baseline motor characteristics of the PIGD patients with diﬀerent DBS response.**

|  | **Good responders**  **(N=24)** | | | **Weak responders**  **(N=14)** | | |
| --- | --- | --- | --- | --- | --- | --- |
| **Motor symptom** | **MedOff** | **MedOn** | **Improvement**  **(%)** | **MedOff** | **MedOn** | **Improvement**  **(%)** |
| **HY** | 2.8 ± 0.5 | 2.3 ± 0.4^a*^ | 0.5 ± 0.6  (15 ± 19%) | 2.6 ± 0.5 | 2.5 ± 0.5 | 0.1 ± 0.4  (3 ± 18%) |
| **MDS-UPDRSIII** |  |  |  |  |  |  |
| Total score | 52.6 ± 13.7 | 23.8 ± 7.8^a**^ | 28.8 ± 9.5  (54 ± 12%) | 46.4 ± 14 | 25.9 ± 5.9^a**^ | 20.4 ± 13.7  (40 ± 20%) ^b*^ |
| Tremor total | 4.7 ± 4.4 | 0.6 ± 1.4^a**^ | 4.1 ± 4.5  (81 ± 33%) | 2.1 ± 2.4 | 0.9 ± 1.5 | 1.2 ± 2.3  (42 ± 50%) |
| Rigidity total | 9.6 ± 4 | 4.9 ± 3.5^a**^ | 4.8 ± 2.7  (52 ± 25%) | 11.6 ± 4.6 | 6.5 ± 3.6^a**^ | 5.1 ± 3.6  (41 ± 28%) |
| Bradykinesia total | 27.2 ± 5.9 | 13.9 ± 4.1^a**^ | 13.3 ± 4  (49 ± 12%) | 23.8 ± 8.2 | 13.7 ± 3.8^a**^ | 10.1 ± 8  (35 ± 29%) |
| Axial total | 11.1 ± 4.5 | 4.4 ± 3.4^a**^ | 6.7 ± 3.2  (62 ± 23%) | 8.8 ± 3.9 | 4.8 ± 2.6^a**^ | 4 ± 3.2  (42 ± 28%) |
| Speech | 1.5 ± 1.1 | 0.8 ± 0.7^a*^ | 0.7 ± 0.8  (46 ± 36%) | 1.3 ± 0.8 | 0.9 ± 0.6 | 0.4 ± 0.5  (24 ± 33%) |
| Arising from chair | 1.4 ± 1.3 | 0.1 ± 0.3^a**^ | 1.2 ± 1.2  (91 ± 25%) | 0.6 ± 1 | 0.1 ± 0.3 | 0.6 ± 0.9  (90 ± 22%) |
| Gait | 2 ± 0.9 | 0.7 ± 0.6^a**^ | 1.3 ± 0.9  (64 ± 34%) | 1.7 ± 0.7 | 0.7 ± 0.5^a*^ | 1 ± 0.7  (55 ± 32%) |
| Freezing of gait | 1.5 ± 1.4 | 0.4 ± 0.7^a*^ | 1.1 ± 1.3  (68 ± 39%) | 1.1 ± 1.5 | 0.2 ± 0.6 | 0.9 ± 1.3  (75 ± 42%) |
| Postural stability | 2.7 ± 1.4 | 1.3 ± 1.4^a*^ | 1.4 ± 1.5  (58 ± 40%) | 2.4 ± 1.4 | 1.9 ± 1.5 | 0.4 ± 1  (14 ± 77%) |

Values are presented as means±SDs.

***a*** indicates the statistical difference of the MDS UPDRS-III score and subscores between the MedOff and MedOn states.

***b*** indicates the statistical difference of the improvement or improvement percentage of MDS UPDRS-III score and subscores between the two groups.

**p* value < 0.05. ***p* value < 0.01.

MDS UPDRS-III, Movement Disorder Society Uniﬁed Parkinson’s Disease Rating Scale-Motor Part; PIGD, Postural Instability and Gait Diﬃculty; MedOff, preoperative off-medication state; MedOn, preoperative on-medication state.

**Supplemental Table 5. Stimulating parameters at the last follow-up.**

|  | Left | Right |
| --- | --- | --- |
| Amplitude (V) | 3.3 ± 0.7 | 3.0± 0.8 |
| Pulse width (µs) | 83.4 ± 13.6 | 81.4 ± 13.6 |
| Frequency (Hz) | 147.8 ± 28.0 | 147.8 ± 28.0 |

**Supplemental table 6. Levodopa equivalent daily dose change after surgery.**

|  | **The whole cohort  (N=55)** | **PIGD (N=38)** | **IND (N=6)** | **TD  (N=11)** |
| --- | --- | --- | --- | --- |
|  |  |  |  |  |
| Preoperative LEDD (mg) | 813.1 ± 280.8 | 825 ± 294.5 | 820.8 ± 246.1 | 767.6 ± 267.8 |
| Postoperative LEDD (mg) | 658.9 ± 282.6^a**^ | 720 ± 294.5^a*^ | 608.8 ± 295.9 | 475 ± 116.3^a**b**^ |
| LEDD change (mg) | 154.2 ± 243.5 | 105 ± 241.8 | 212 ± 246.8 | 292.6 ± 202.9 |
| percentage LEDD change | 16 ± 32 % | 8.4 ± 33.1 % | 28.1 ± 33.4% | 35.2 ± 13.6 % ^b**^ |

*a* indicates the statistical difference of LEDD between postoperative and preoperative situations.

*b* indicates the statistical difference of LEDD change comparing to PIGD subtype.

**p* value < 0.05. ***p* value < 0.01.

PIGD, Postural Instability and Gait Diﬃculty; TD, tremor dominant; IND, intermediate; LEDD, Levodopa equivalent daily dose.
